# Enhanced lipid and starch productivity of microalga (*Chlorococcum* sp. *TISTR 8583*) with nitrogen limitation following effective pretreatments for biofuel production

**DOI:** 10.1016/j.btre.2018.e00298

**Published:** 2018-12-18

**Authors:** Zia Ur Rehman, Anil Kumar Anal

**Affiliations:** a Food Engineering and Bioprocess Technology, Department of Food, Agriculture and Bioresources, Asian Institute of Technology, P.O. Box 4, Klong Luang, Pathumthani, 12120, Thailand; b Institute of Biochemistry, University of Balochistan, Sariab Road Quetta, Balochistan, Pakistan

**Keywords:** Microalgae, Pretreatments, Biofuel, Starch, Lipids

## Abstract

The study was conducted to evaluate the conditions to enhance the accumulation of lipids and starch in *Chlorococcum* sp. *TISTR 8583* for the production of biofuel. The *Chlorococcum* sp. *TISTR 8583* was cultivated on BG-11 medium under optimized light intensity. The nitrogen limitation (NL) enhanced the accumulation of both starch and lipids and resulted in 34.02% total sugars as compared to 22.57% on nitrogen supplemented (NS) media only. Similarly, the nitrogen supplemented (NS) media produced 17.05% lipids as compared to 29.59% lipids by NL media. The biomass was investigated for biodiesel and bioethanol production by adopting different pretreatment strategies, such as enzyme, acid and alkaline pretreatments. The alkaline pretreatment was found to be efficient strategy (23.67 wt% sugars/g algal biomass: 1.2% (w/v) at 140 ⁰C for 30 min) while the acid pretreatment (1%: v/v; 140 °C) was least effective pretreatment strategy with the yield of 14.83 wt% sugars/g algal biomass.

## Highlights

• To enhance the lipid and starch productivity of *Chlorococcum* sp. *TISTR 8583* for biofuel productions.
• To identify suitable pretreatment strategy for release of fermentable sugars and lipids extraction from algal biomass.
• To convert the released fermentable sugars and lipids into bioethanol and biodiesel respectively.

## Graphical abstract

**Figure:**
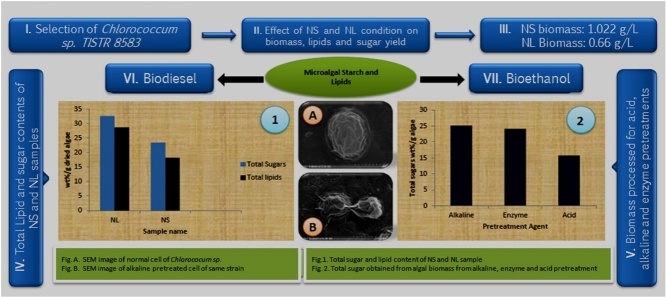

## 1 Introduction

The depletion of fossils fuels due to rapid global development with respect to its environmental problems needs to be replaced by renewable and sustainable fuel resources [1]. The first generation biofuel has clash with global food security whereas, second generation biofuels seem economically infeasible, and lead to the deforestation. In this scenario, the energy demands need to be fulfilled through renewable resources to reduce the risks of environmental pollution and global food security. In most of the developing countries, the agriculture land is used for growing oil producing crops for biofuel production. The third generation biofuel makes sense by its special features such as, having no direct impact on global food security, environmental problems and its year round production.

The two types of biofuels (biodiesel and bioethanol) are produced from oil crops [2] and sugar producing crops [3] respectively while the microalgae is a single platform for both types of biofuels due to the production of both biological components (starch and lipids). The conversion of lingo-cellulosic material is expensive because of lignin and hemicelluloses contents which reduce surface area for enzyme access to the cellulose contents hence production cost is found to be very high. Its removal needs sophisticated technology which makes it unfeasible, while in case of microalgae, the absences of lignin and hemicelluloses contents make it best cellulosic feedstock for biofuel production.

Microalgae contain starch, cellulosic materials without lignin and oil in its cellular matrix, which can be modified to produce biofuels. Most of the microalgal species contain more than 37% starch contents, which make them possible to explore for bioethanol production [4]. The endogenous starch and oil can be manipulated to enhance by varying the nutrients in growth media and conditions. The microalgal strains such as *Dunaliella, Scenedesmus, Chlorella, Spirulina* and *Chlamydomonas* are reported to contain abundant amount of starch and other carbohydrates (more than 50% of dry weight), which is used as feedstock for production of ethanol [5]. Yao et al. [6] reported an increase in total starch contents of microalga *Tetraselmis subcordiformis* to 62.1% (dry weight). Bush and Hall [7] used *Cladophoraceae, Zygnemataceae, Oedogoniales* or their co-cultures for production of ethanol (US Patent 7,135,308).

The *Chlorococcum* sp. *TISTR 8583* was obtained from Thailand Institute of Science and Technology Research Centre (TISTR), Bangkok, Thailand. *Chlorococcum* sp. is a chlorophycean and single celled fresh water green alga, with a capability of potential accumulation and storage of lipids inside the cell [8]. It is distributed across wide range of terrestrial and aquatic habitats [9]. The cell structure of *Chlorococcum* sp. *TISTR 8583* is ellipsoidal (with varying size and rough cell wall) and is solitary in nature with thin mucilage as reported by Watanabe and Lewis [10]. In their research study, Kirrolia et al [11] investigated the *Chlorococcum* sp. strain with highest biomass yield (0.95 g/L) in BG-11 medium. Furthermore, the greater biomass yield and lipid accumulation (27%) was observed by Aravantinou and Manariotis [12] cultivating *Chlorococcum* sp. under artificial light as compared to direct sunlight. The *Chlorococcum* sp. *TISTR 8583* contains 26% starch content [13]. Additionally, the *Chlorococcum* sp. has been investigated for excellent self flocculating nature which facilitates the easy biomass harvest as well as removal efficiency of sulfur and nitrogen from waste water as bioremediation agent [14].

This particular strain has not yet been reported to enhance the accumulation of starch and lipids by nutrient limitation (nitrogen limitation) and varying light conditions (optimum biomass yield) for co-production of biodiesel and bioethanol with different pretreatment methods. In this study, *Chlorococcum* sp. *TISTR 8583* was investigated for two of its major contents in the forms of starch and lipids (as this strain has not yet been explored for the total concentration of lipid and its fatty acid composition linked with biodiesel quality). First of all, the strain was tested for the enhanced accumulation of both lipids and starch contents by nitrogen stress condition and microalgal carbohydrates and lipids were extracted and converted to bioethanol and biodiesel respectively (for the first time with this specific strain).

## 2 Materials and methods

### 2.1 Algal cultivation and biomass

The *Chlorococcum* sp. *TISTR 8583* was purchased from TISTR, Thailand, inoculated and grown in pre-specified media in one-liter Erlenmeyer flasks containing BG-11 and supplied with continuous aeration at 14:10 light dark cycle. The light intensity was adjusted to 4.34 klx (Illumination meter: INS DX-200). The pH of the medium was adjusted to 6.7 by addition of 1 M acetic acid (the amount of acetic acid was negligible and used for adjusting pH only and was not used to promote heterotrophic growth as an organic carbon source for algal culture system). The growth was monitored by measuring the optical density (OD) at 680 nm, using UV spectrophotometer (Unicam UV/Visible spectrometer). Harvested Biomass was dried overnight in oven at 75˚C and measured as g/L. The results were verified by triplicate batches.

### 2.2 CHN analysis

The total carbon, hydrogen and nitrogen (C: H: N) ratio of dried nitrogen limited (NL) and nitrogen supplemented (NS) algal powder was analyzed by CHNS (Micro) analyzer (Leco Truspec). The samples were burnt at 1100 °C under O_2_ atmosphere by using helium as carrier gas.

### 2.3 Chemical finger-printing of functional groups of algal biomass through fourier transform infrared spectroscopy (FTIR)

The algal sample was dried in an oven (Memmert: Model: 600, GmbH Co, Ltd) at 70 °C overnight. The dried biomass was ground to fine powder. The powder mixture was transferred to compression dye under high pressure to form a pellet. The pellet was kept in sample cuvette and analyzed according to standard FTIR (Perkin Elmer) test method ASTM: 1252-98 with light source in middle range infrared (4000–600cm^−1^). The technique was employed for the determination of chemical composition of sample based on functional groups.

### 2.4 Cell disruption by ultrasonication

The harvested algal cells were disrupted by ultrasonicator (Branson, USA) in order to release the oils, starch and fermentable sugars from cells via acid, alkaline and enzymatic pretreatment approaches. Cellulase enzyme was specifically used to digest the microalgal cell wall in order to make the cells more susceptible to disruption. The frequency of ultrasonic machine was 47 KHz for acid, alkaline and enzyme pretreatment of pre-specified algal biomass at 100 °C for 30 min.

### 2.5 Oil extraction, trans-esterification and FAME analysis by GC–MS

All samples obtained from different nutrient conditions, were subjected to oil extraction by following Folch method with slight modification [15] and were converted to biodiesel by trans-esterification in presence of alkali catalyst. Briefly, the fatty acid methyl esters (FAME) were analyzed by GC–MS (Agilent Technologies. USA). The GC–MS consisted of Agilent Technologies GC-7890 A chromatograph and an Agilent Technologies 5975C mass spectrophotometer equipped with DB-WAX: 1227032 capillary column with a length of 30 m, an internal diameter of 0.25 mm and a film thickness of 0.25 um. For analysis of fatty acid methyl esters (FAME), the oven temperature was kept 40 °C for 5 min then programmed at 5 °C per min to 250 °C and was maintained for 10 min. The split injection was carried with 100:1 split ratio and the Injector temperature was 250 °C. The helium gas was used as mobile phase and the flow rate was 9.63 ml/min. The interface temperature and ionization mode of integrated MS system were 230 °C and EI respectively while electron energy was 70 eV with full scan acquisition mode and mass range of 35–750 amu.

### 2.6 Pretreatment strategies

The acid and alkaline and enzyme pretreatments were conducted for NL samples for the first time with this strain (*Chlorococcum* sp. *8583*). The acid and alkali pretreatments were carried by testing different parameters such as acid and alkali concentrations (acid: 0.50%, 0.80%, 1%, 1.5% (v/v) and alkali: 0.50%, 1%, 1.2%, 2% w/v) and pretreatment temperatures (100 °C, 120 °C, 140 °C and 160 °C). The samples were kept in oven for 15–50 min and were followed by cooling for 30 min at room temperature (25 °C). The acid pretreated samples were sonicated (47 KHz) (Ultrasonicator: Branson, USA) at 100 °C for 30 min further in order to disrupt the cells. The biomass containing mixtures were further centrifuged (Hsiangtai, model: CN-10001) at 4500 rpm for 10 min and were filtered by whatman filter paper #1, by using vacuum filtration for obtaining pure hydrolysate solution.

The samples were analyzed by phenol sulphuric method [16,17] for total sugar contents. The remaining fermentable solution was refrigerated at -20 °C for further use. The cellulase enzyme pretreatment was carried by 0.01 g/g, 0.015 g/g and 0.02 g/g cellulase at different pH values (3.8, 4.8 and 5.8) and different temperature ranges (25 °C, 45 °C and 65 °C). The optimum enzyme concentration, pH and temperature were noted. The amylase pretreatment was conducted by adding 0.005% (w/w) α-amylase (from *Aspergillus oryzae* (Sigma-Aldrich) at pH 4.5 and 90 °C for 30 min in water bath. The samples were treated with 0.2% (v/w) amyloglucosidase enzyme from *Aspergillus niger* (Sigma-Aldrich) at 55 °C for 30 min [18]. The final digested samples were analyzed by sulphuric acid method and HPLC system. The results were conducted in triplicates.

The pretreated *Chlorococcum* sp. *TISTR 8583* cells were examined under scanning electron microscope (SEM: Hitachi S-3400 N Japan) for overall cell damage caused by acid, alkaline and enzyme pretreatments.

#### 2.6.1 Algal biomass liquefaction, saccharification and HPLC analysis

The monosaccharides were obtained from combined strategy of enzyme pretreatment (cellulose enzyme at 45 °C, 0.015 g/g enzyme and pH 4.8 for 72 h) and the post-enzyme alkaline pretreatment at pre-optimized conditions (1.2% NaOH at 140 °C for 30 min). The pretreated samples were further incubated for liquefaction of algal starch with thermostable α-amylase. The liquefaction of algal starch was carried at optimum conditions (0.005% enzyme, 90 °C, 30 min) as reported by Choi et al. [18]. After completion of liquefaction process, the partially digested samples were further digested by starch saccharifying enzyme, amyloglucosidase during saccharification process at pre-reported optimum conditions (0.2% enzyme, 55 °C, 30 min and pH 4.5) by Choi et al. [18].

HPLC analysis was performed by Agilent system (HP 1100 Agilent USA) for determination of glucose concentration in above mentioned enzymatically digested algal samples, using RID detector (Agilent) and the system consisted zorbax carbohydrate column (4.6 × 150 mm × 5 μm), the column temperature was held constant at 40 °C. The mobile phase used was acetonitrile: deionized water at the ratio of 75:25.

### 2.7 Measurement of total sugars

The phenol sulphuric acid method [16] was adopted to measure the sugar contents with slight modifications. Dried sample (10 mg) was dissolved in 50 ml distilled water and kept in ultrasonicator (47 KHz) at room temperature for 30 min in order to dissolve the biomass. Each microalgae solution (1 ml) was mixed with one ml of 5% (w/v) phenol solution and 2.5 ml of concentrated sulphuric acid and incubated at room temperature (25 °C) for 30 min. The color of the mixture turned orange and was determined by UV- spectrophotometer (Unicam UV/Visible spectrometer) at 490 nm.

### 2.8 Ethanol fermentation by yeast

The *saccharomyces cerevisiae* was inoculated in LB (Luria Broth) medium aerobically for 48 h as reported by Harun et al. [45] and the sample was centrifuged at 2500 rpm for 5 min in sterilized tubes for removal of nutrient rich LB media for anaerobic fermentation. The supernatant was discarded and the yeast was reconstituted in hydrolysates medium and transferred to the fermentation tube, which was kept in incubator at 30 °C for 48 h. The fermented samples were analyzed by GC-MSD (Gas chromatography- mass selective detector).

### 2.9 Ethanol measurement by GC/MSD

The ethanol produced by *saccharomyces cerevisiae* from algal hydrolysates, was analyzed by GC/MSD system (GC 6890N-MS-D 5973 Head space-7694 Agilent Technologies USA). The condition of analysis was 200 °C oven temperature with increase of 20 °C per minute. The flow rate of sample was kept 1.0 ml/min. The head space oven temperature was kept 70 °C while the sample valve temperature was 140 °C. The transfer line temperature was 150 °C. The sample

(5.0 μL) was injected to the system for analysis. The detector used was MS with scan 20–350 m/z. All fermented samples were analyzed by iodofrom reaction (qualitative test for presence of ethanol in test samples) for potential samples to be analyzed by GC-MSD.

## 3 Results and discussions

### 3.1 Growth conditions and cell composition

The BG-11 fed batch (100 ml) was first inoculated (5% inoculum in 250 ml Erlenmeyer flasks) in incubator shaker (ES: Edmund Buhler: Model: TH 25) and were further cultured in one liter Erlenmeyer flasks for 18 days under constant irradiance (14:10 light: dark cycle) and constant air supply (Air compressor: Hailea, Model: ACO-318) for optimization study. The *Chlorococcum* sp. *TISTR 8583* was first optimized on light intensity by cultivating it on three different light intensities, Low irradiance (LI), Medium irradiance (MI), and high irradiance (HI). The MI (4.34 klx) was found to be optimum light intensity for optimum algal growth. After light intensity optimization, the strain was subjected to NS and NL media for enhancing the lipid and starch contents. The selected strain produced high biomass (1.02 g/L) in case of nitrogen supplemented (NS) media as compared to nitrogen limited media (0.66 g/L). The oil contents produced by *Chlorococcum* sp. *TISTR 8583* were found to be 17.05% (lower) in nitrogen supplemented (NS) samples with an increase to 29.59% (12.54% increased) in nitrogen limited (NL) samples while the carbohydrate contents in NS samples were 22.57% as compared to 34.02% (11.5% increased) in nitrogen limited (NL) samples (Table 1) which we report for the first time with this specific strain. In one of the study, *Chlorella vulgaris* [19] produced 60% starch (dry weight) under sulfur deprivation and high irradiance. In a most recent study, Xie et al. [44] obtained 0.33 g/L biomass with nitrogen limited media while maximum biomass yield of 0.78 g/L from *Chlorella sorokiniana* supplemented with 2 g/L glycine (costly) concentration in nutrient media which decreased the lipid accumulation due to the presence of excess nitrogen (in the form of glycine) and increases the total input cost of the culture system to make it economically infeasible hence in our case the biomass yield was higher in both cases using BG-11 without adding any organic source such as glycine.

**Table 1:** Cell composition of NS and NL samples of *Chorococcum sp*. TISTR 8583.

Contents	NS Sample (%)	NL sample (%)
Total sugars	22.57 ± 1.26	34.02 ± 1.66
Glucose	54.52	–
Total lipids	17.05 ± 1.03	29.59 ± 0.82
Moisture contents	4.44 ± 0.26	3.55 ± 0.22
Others	55.94	32.84

The correlation of sugar and lipid concentrations with biomass yield (sugar and lipids accumulation vs biomass per liter media) in both NL and NS samples was evaluated and nitrogen limitation (NL) was found to be relatively and competitively better strategy than nitrogen supplementation (NS). The final quantity of sugar content in both NL and NS samples remained almost equal due to lower biomass yield (0.66 g /L) in NL sample in per liter BG-11 nutrient media i.e. 0.225 g/L (0.225 g/0.66 g dried algae/L) and 0.2257 g/L (0.2257 g/1 g dried algae/L) in NL and NS samples respectively while the lipid content in NL and NS samples was 0.1953 g/L (0.1953 g/0.66 g dried algae/L) and 0.1715 g/L (0.1715 g/1 g dried algae/L) respectively. The NL produced 0.024 g/L higher lipid content per liter than NS strategy. Based on the evaluation of sugar and lipid concentrations versus biomass yield per liter nutrient media in both NL and NS samples indicated that the NL strategy is relatively good for increased accumulation of lipids/L even with 33% less biomass (0.66 g/L) as compared to lipid /L in NS biomass (1.022 g/L). The relatively higher lipid yield per liter in NL media (0.0247 g/L) coupled with the cost effectiveness due to 3 times (66.66%) less nitrogen source utilization (0.5 g/L NaNO_3_) in NL media in comparison to higher nitrogen source utilization (1.5 g/L NaNO_3_) in NS media make NL strategy as best option as compared to NS strategy for algal fuel production.

The moisture contents were 4.46% and 3.55% in NS and NL samples respectively (Table 1). The decrease of moisture contents in NL sample may be due to hydrophobic interaction of increasing lipid contents with polar water molecules in cell cytosol. The more the lipid contents, the more likely the repulsion among polar water molecules and non-polar lipids.

The carbon, hydrogen and nitrogen ratio (C: H: N) of *Chlorococcum* sp. *TISTR 8583* was analyzed as an alternative evidence for the effect of nitrogen limitation on the enhanced accumulation of lipids and carbohydrates. The C: N ratio (%) was found to be 51.5: 4.5 in NL sample as compared to 42.4: 5.9 of NS samples. The decrease in nitrogen contents (4.5 in NL as compared to 5.9 in NS) indicated the reduced protein contents while the increase in total carbon (51.5 in NL as compared to 42.4 in NS) is an alternative evidence of enhanced accumulation of lipids and carbohydrate contents. Dorling et al. [20] also found similar results by measuring carbon/ nitrogen (C/N) ratio in symbiotic *Chlorella* under changing pH environment.

### 3.2 FTIR analysis

The powdered samples were examined in the middle range infrared region (4000–600 cm ^−1^) by Perklin Elmer spectrophotometer system which gave characteristic peaks and their relevant functional groups. Different peak ratios were obtained for different functional groups present in *Chlorococcum* sp. *TISTR 8583.* The results confirmed the presence of functional groups of alcohols, ketones, alkanes, aldehydes, amides, carboxylic acids, halogens, epoxides and sulfur compounds. The FTIR is useful technique in quantifying total lipids and carbohydrates for –C=O and —OH stretching. The range of 3296 cm ^-1^ stretching was due to −OH group, which corresponds to carbohydrates while 2957 cm ^−1^ to 2853 cm ^−1^ corresponds to C–H vibrations mostly indicating methylene group of lipids. The unsaturated C–H stretching was observed in the region of 3011 cm ^−1^ for unsaturated bonds in long chain fatty acids. The N—H stretching was noticed on 3296 cm ^−1^. The region of 3083 cm ^−1^ was assigned to compounds of unknown origin while the region of 1743 cm ^−1^ was O= C–O bond stretching which corresponds to esters of fatty acids. The IR region of 1644 cm ^−1^ and 1549 cm ^−1^ peaks described the C=O bonds of amide I and amide II linkages respectively which corresponds to proteins. The region of 1456 cm ^−1^ corresponds to CH_2_ and CH_3_ bending in long chain fatty acids and proteins. The region of 1155 cm ^−1^ and 1032 cm ^−1^ is associated with aliphatic compounds.

### 3.3 Strategy to enhance lipid accumulation and FAME Analysis

**Fig. 1:**
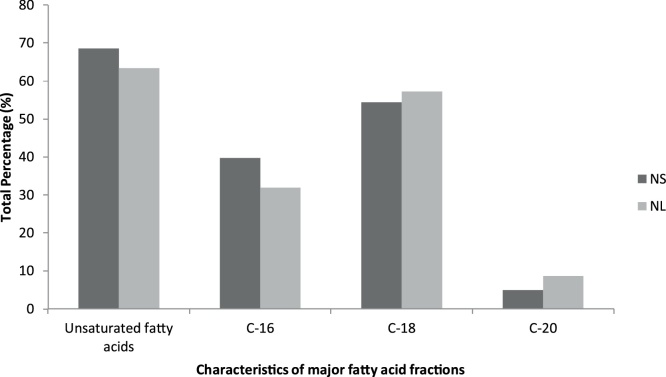
Characteristics of major fatty acid fractions (%) of NS and NL samples of *Chlorococcum* sp. *TISTR 8583*.

The microalgal oil was trans-esterified and analyzed by GC–MS. Selected strain produced 17.05% and 29.59% lipids (12.54% increased) on nitrogen supplemented (NS) and nitrogen limited (NL) media respectively. Liu et al. [21] has reported few potential microalgal strains having to produce high biomass productivity and enhanced lipid contents. The cultivation of microalgal strains under nitrogen stress enhances the lipid contents [22,23]. In this study the *Chlorococcum* sp. *TISTR 8583* was grown on nitrogen supplemented and nitrogen limited BG-11 cultivation media for 18 days. The most abundant fatty acid was 9-octadecenoic acid (C-18) was 26.17% in NS and 23.24% in case of NL samples (Table 2). The next abundant fatty acid was found to be hexadecenoic acid (20.7%) in nitrogen supplemented sample while 19.4% in NL sample. An increase in octadecanoic acid (11.25%), 9,12,15-Octadecatrienoic acid (4.27%) and 5,8,11,14-Eicosatetraenoic acid (6.23%) was observed in NL samples as compared to NS samples (Table 2). The nutrient deficient conditions were found to be effective for enhanced accumulation of lipids as well as enhancing the production of C-18 and C-20 fatty acids. The strain produced 68.2% and 63.38% unsaturated fatty acid in NS and NL samples respectively (Fig. 1). Many previous studies have shown that the nitrogen limitation triggers the accumulation of lipids in algal cells [[24], [25], [26]]. The selected strain was observed as good choice for biodiesel production.

**Table 2:** The fatty acid profile of NS and NL samples of *Chorococcum sp*. TISTR 8583 analyzed by GC–MS.

Name of fatty acid	NS %	NL %
Tetradecanoic acid (14:0)	0.312	0.409
Isopropyl myristic acid	–	0.232
Nonadecanoic acid (19:0)	–	0.231
Pentadecanoic acid (15:0)	0.162	0.173
Hexadecanoic acid (16:0)	20.71	19.398
Palmitoleic acid (16:1)	1.765	1.323
7,10-Hexadecadienoic acid (16:2)	3.8	2.60
Heptadecanoic acid (17:0)	0.215	0.2
7,10,13-Hexadecaenoic acid (16:3)	4.138	4.5
5,8,11,14,17-EPA (20:5)	0.836	0.74
Octadecanoic acid (18:0)	2.725	11.25
9-octadecenoic acid (18:1)	26.171	23.24
Linoleic acid (18:2)	11.567	9.27
6,9,12,15-Octadecatetraenoic acid (18:4)	0.587	1.81
9,12,15-Octadecatrienoic acid (18:3)	0.545	4.27
5,8,11,14-Eicosatetraenoic acid (20:4)	2.160	6.23
11-Eicosaenoic acid (20:1)	0.816	0.625
7,12-Pentadecenoic acid (15:2)	–	0.6

The FAME profile is the key to determine fuel properties of samples under investigation. The occurrence of C16 and C18 fatty acid methyl esters give good properties for biodiesel [27]. The selected samples showed 92.6% of C16 to C18 fatty acids which optimize the relationship between cold flow properties and oxidative stability [27]. These research findings suggest that *Chlorococcum* sp. *TISTR 8583* oil can have low iodine value and high cetane number which qualifies the US (ASTM D6751) standards [28].

### 3.4 Algal biomass liquefaction, saccharification and HPLC analysis

In HPLC analysis, the main monosaccharide detected in NS sample was glucose with 54.5% concentration (Table 1), obtained from combined strategy of enzyme pretreatment (cellulose enzyme at 45 °C, 0.015 g/g enzyme and pH 4.8 for 72 h) and the post-enzyme alkaline pretreatment at pre-optimized conditions (1.2% NaOH at 140 °C for 30 min). The pretreated samples were then incubated for liquefaction of algal starch with thermostable a-amylase which randomly hydrolyze alpha-(1-4) glyosidic linkage in starch molecule [29] to produce limit dextrin molecules with three or alpha-1-4-linked glucose residues [30]. During liquefaction process, the starch granule is converted into a partially digested starch solution with low viscosity and increased surface area for further hydrolysis to monosaccharides by amyloglucosidase enzyme. The liquefaction of algal starch was carried at optimum conditions (0.005% enzyme, 90 °C, 30 min) as reported by Choi et al. [18]. After completion of liquefaction process, the partially digested samples were further digested by starch saccharifying enzyme, amyloglucosidase which catalytically hydrolyze the alpha-(1e)- and alpha-(1nd)- glycosidic bonds of liquefied dextrin molecules. Amyloglucosidase enzyme during saccharification process at prereported optimum conditions (0.2% enzyme, 55 °C, 30 min and pH 4.5) by Choi et al. [18].

### 3.5 Enzymatic pretreatment

**Fig. 2:**
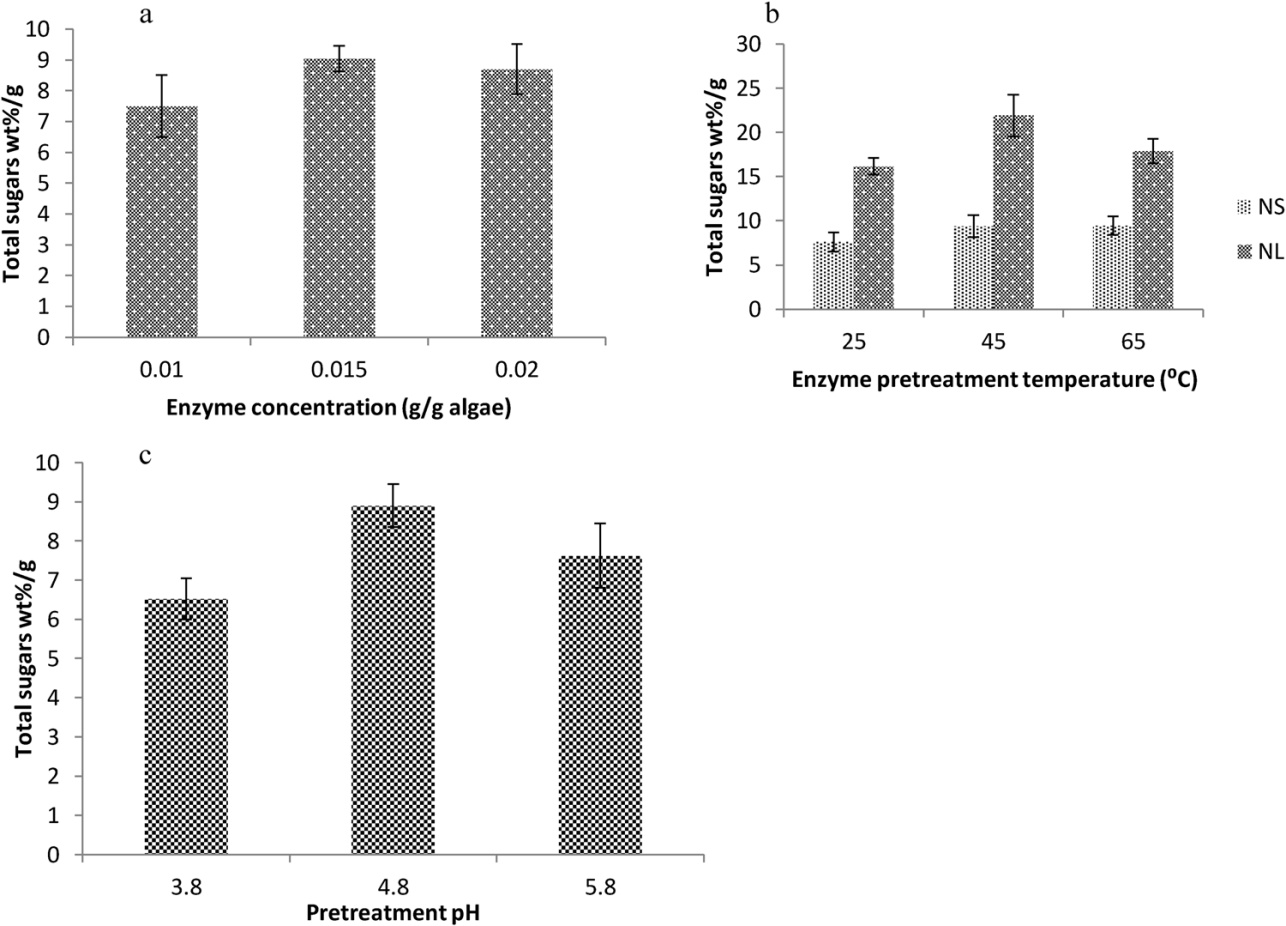
Effect of different enzyme concentrations, temperatures and pH on sugar yield **a.** Cellulase enzyme pretreatment of NS and NL samples of *Chlorococcum* sp. *TISTR 8583* for total sugar contents at different temperature ranges. **b.** pH study of cellulase enzyme for cell wall digestion of *Chlorococcum* sp. *TISTR 8583*.**c.** Concentration of enzyme used in cell wall digestion of *Chlorococcum* sp. *TISTR 8583* (results were conducted in triplicates).

Pretreatment of microalgal biomass (both NS and NL) with cellulase enzymes of 0.01, 0.015 g/g and 0.02 g/g concentrations was carried at different pH (4.2, 4.8 and 5.8) and ranges of temperature (25 °C, 45 °C and 65 °C). The pre-determined quantity of biomass (10 mg) was added to 10 ml of acetate buffer and cellulase enzyme solution. The samples were kept at different temperatures for 72 h. The optimum enzyme activity was observed with 0.015 g/g enzyme at pH 4.8 and 45 °C (Fig. 2a)

The enzymatic pretreatment seems better strategy in order to digest the microalgal cell wall at optimum conditions. The cell wall digestion followed by ultrasonication releases starch and made access to amylase activity for further hydrolysis to fermentable sugars. The highest yield of sugars by NL samples was 21.94 wt% sugars/g algae at 45 °C (0.015 g/g enzyme, pH 4.8) for 72 h while the lowest sugar concentration was observed at 25 °C with the same physical conditions. As compared to enzyme pretreatment, the alkaline and acid pretreatment generated 23.67 wt% sugars/g algae (best strategy of all) and 14.83%wt sugars/g algae (least effective) respectively. Similar results were obtained by Harun and Danquah [31] by using cellulase pretreatment of *Chlorococcum humicola* and obtained maximum fermentable sugars by using 0.02 g/g celllulase enzyme at pH 5.5 and 40 °C. The cell wall digestion of *Chlorococcum* sp. *TISTR 8583* was examined under scanning electron microscope with large cuts on the cell surface (Fig. 5c).

The optimum hydrolysis temperature was 45 °C, reflecting the highest glucose yield (Fig. 2b). Temperatures below and above 45 °C resulted in lower yield. Saha and Cotta [32] and Mtui [33] have reported the optimum temperature range of 30–45 °C for cellulase activity, obtained from *Trichoderma reesei.* An increase in temperature affected the process of hydrolysis by increasing molecular collisions between enzyme and substrate molecules which results in accumulation of heat to denature the enzyme [34]. The optimum pretreatment pH was found to be 4.8 (Fig. 2c)

### 3.6 Alkaline pretreatment

**Fig. 3:**
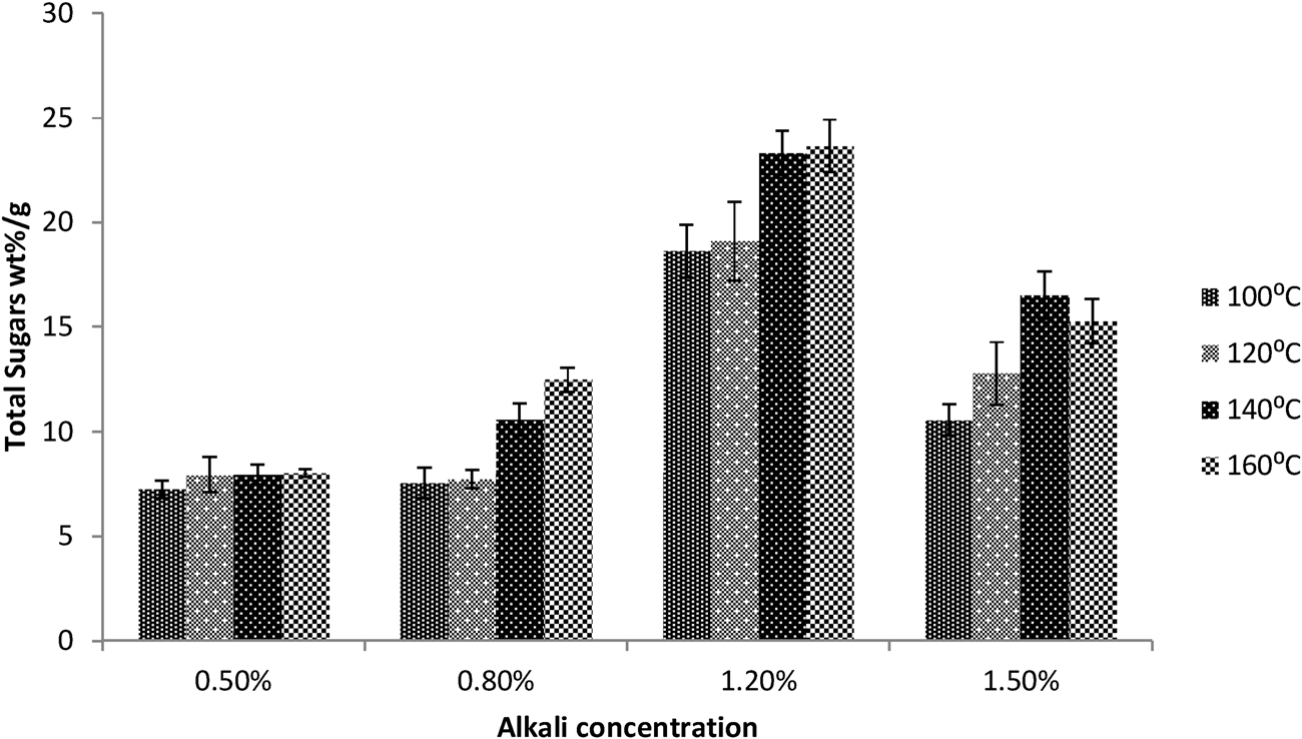
Alkaline pretreatment of NL samples for total sugar contents at different alkali concentrations and temperature ranges. All results were conducted in triplicates.

The microalgae do not have lignin in their cellular matrices, hence the alkaline pretreatment also seems efficient strategy for release of fermentable sugars stored inside the algal cell walls [1]. The alkaline pretreatment was the most efficient chemical pretreatment process in releasing fermentable sugar. As shown in SEM micrograph (Fig. 5b), alkaline pretreated cells were totally punctured which indicates the release of internal matrix from the cells. The highest fermentable sugars released were 23.67 wt% sugars/g algae obtained with 1.2% NaOH at 140 °C for 30 min (Fig. 3), while remained the same at elevated temperature (160 °C). Similar results were obtained by Harun et al. [1] with *Chlamadomonas reihhardtii* biomass (26.1 wt% (g ethanol/g dried algae) at 1% NaOH at 120 °C for 30 min. The elevated temperature and high NaOH concentrations needed in case of *Chlorococcum* sp. *TISTR 8583* may be due to the cell structural differences from *Chlamadomonas reinhardtii.* The lowest glucose was obtained with 0.5% NaOH (7.25 wt% sugars/g dried algae) at 100 °C for 30 min. In another research study, Sivaramakrishnan and Incharoensakdi [35] digested *Scendesmus* sp. via alkaline pretreatment and obtained the maximum yield of sugar with 0.3 N NaOH at 80 °C and 20 min.

### 3.7 Acid pretreatment

**Fig. 4:**
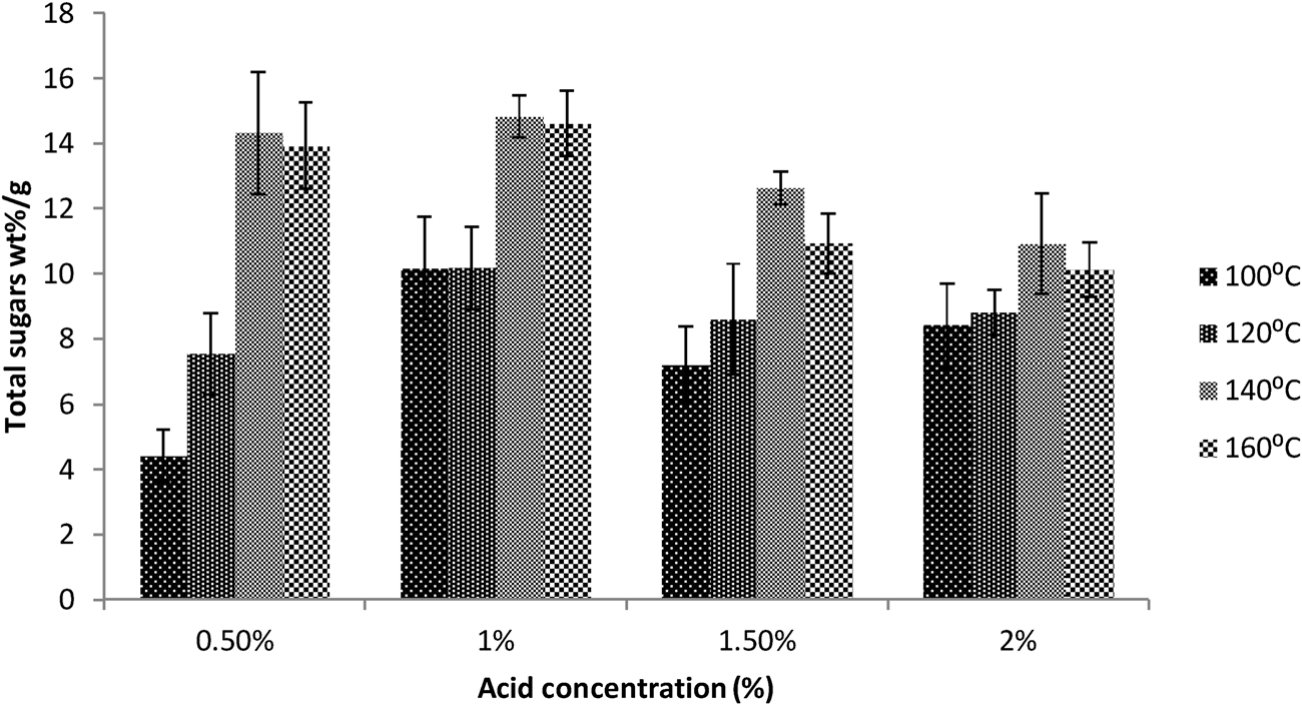
Acid pretreatment of NL samples for total sugar contents at different acid concentrations and temperature ranges. All results were conducted in triplicates.

**Fig. 5:**
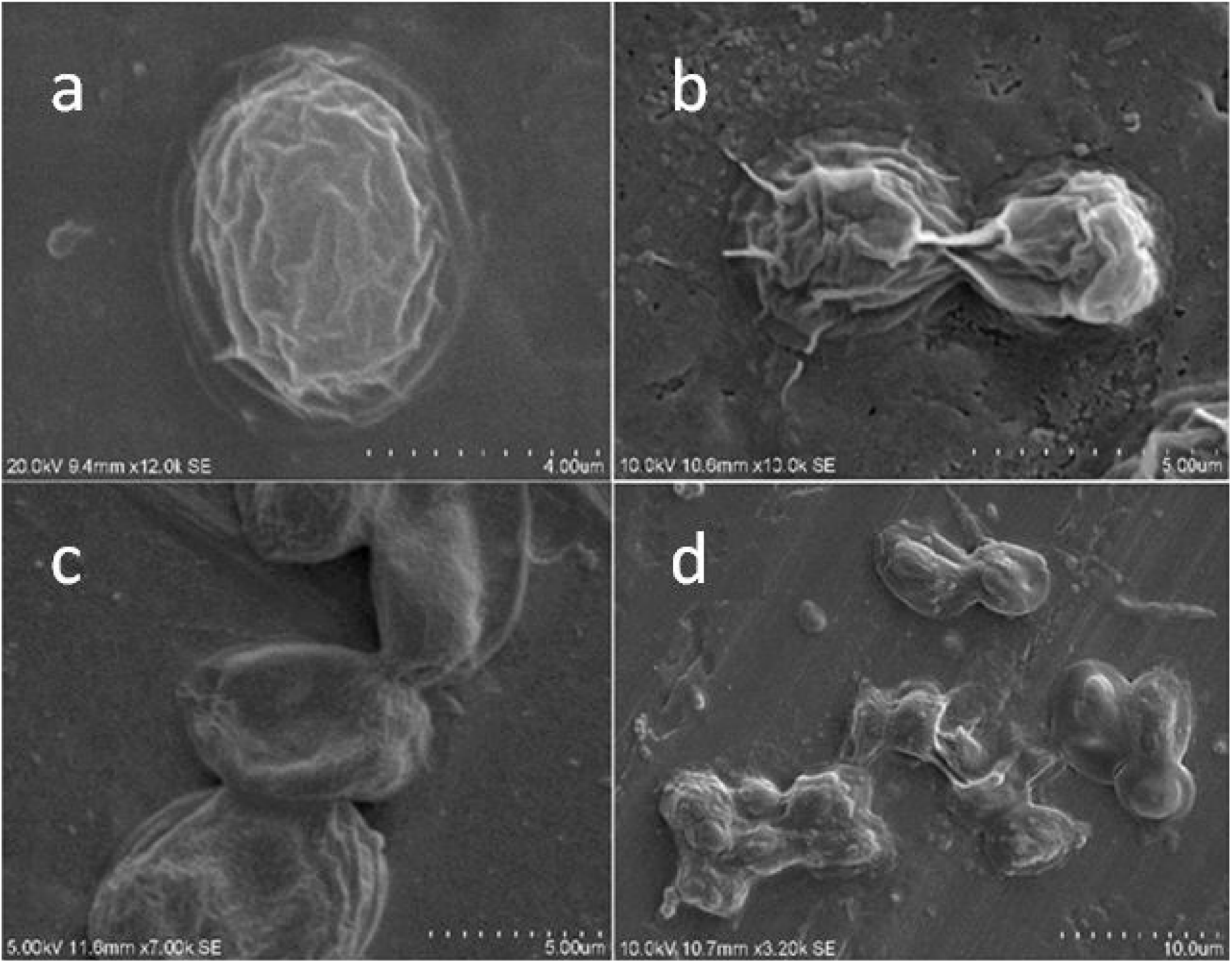
Scanning Electron micrographs (SEM) of: **a.** Normal cells of C*hlorococcum* sp. *TISTR 8583*. **b.** Alkaline pretreated cells of *Chlorococcum* sp. *TISTR 8583*. **c.** Enzyme pretreated cells of *Chlorococcum* sp. *TISTR 8583*. **d.** Acid pretreated cells of *Chlorococcum* sp. TISTR 8583.

The acid pretreatment was the least effective pretreatment strategy as compared to enzyme and alkaline pretreatments. The highest sugars (14.83%wt sugars/g algae) were released by 1% sulpuric acid at 140 °C (Fig. 4). Similar results were obtained by Harun and Danquah [31] with higher bioethanol concentration of 7.2 g/L by loading 15 g/L with 1% acid at 140 °C. In case of this study, the higher temperature (160 °C) has negative effect on the release of fermentable sugars upon high temperature treatment. The higher percentage (1.5%) of sulphuric acid was the least advantageous as the cells were flocculated at higher temperature environment in case of higher sulphuric acid concentration which was confirmed by scanning electron microscopy (Fig. 5d). Similar results were observed by Shokrkar et al. [36] in which higher sulphuric acid and hydrochloric acid concentration (above 2 M) resulted in lower sugar yield (82–84% after 40 min pretreatment as compared to the optimum sugar yield of 94% with 2 M HCl or mixture of 0.5 M H_2_SO_4_) possibly due to sugar degradation as reported by Miranda et al. [37].

Another indicator for explanation of this lower sugar yield phenomenon maybe the conversion of fermentable sugars into hydroxymethylfurfural (HMF). The acid pretreatment with high acid concentration and elevated temperature (140–160 °C) degrades fermentable monosaccharides (hexoses) into hydorxymethylfurfural (HMP) which reduces the level of fermentable sugars in the samples under investigation. Wrigstedt et al. [38] obtained 85% HMF with 0.05 M H_2_SO_4_ using 0.15 g hexose monosaccharide at 160 °C pretreated in microwave hence the measurement of HMF concentration in acid pretreated samples will indicate the intensity of loss of fermentable sugars or reduction of fermentable sugars level.

The least fermentable sugars (4.41 wt% sugars/g algae) were released with 0.5% sulphuric acid treatment at 100 °C for 30 min. The study suggests that the high temperature has inverse relation with acid concentrations. An increase in acid concentration causes flocculation of cells together at higher temperature. The flocculation of cells reduces the surface area for acid activity hence most of the entrapped fermentable sugars remain inside the microalgal cells. The study confirmed that the acids work better in lower concentrations. Similar results were obtained by Del Campo et al. [39] by treating agri-waste with low acid concentrations while the rye straw released high quantity of fermentable sugars [40]. The intracellular components such as carbohydrates and lipids can be converted to bioethanol [18]. Ballesteros et al. [41] also reported the importance of correlation of pretreatment temperature with dilute acids during pretreatment of Bermuda grass and rye straw, which was confirmed by Sun and Cheng [40].

### 3.8 Ethanol production by yeast

The microalgal starch was further evaluated for the production of bioethanol [42]. The acid, enzyme and alkaline pretreated samples were fermented with *Saccharomycese cervisiae*. The pretreated samples were digested with thermostable α-amylase enzyme based on provisional optimum values (0.005% enzyme, 90 °C, 30 min, pH 4.5) as reported by Choi et al. [18]. The samples were further digested by amyloglucosidase (0.2% enzyme) activity at 55 °C for 30 min. The fully digested samples were stored at -20 °C for fermentation. The fermentation was carried with *Saccharomyces cerevisiae* at 30 °C in incubator for 48 h. The ethanol was qualitatively analyzed by iodofrom reaction in order to determine the potential samples for GC-MSD analysis. The highest ethanol was 1.9 g/L obtained from enzyme pretreated samples by loading 10 g of dried algae followed by 1.4 g/L alkaline pretreated samples with the same quantity of biomass used as shown in Table 3. The least ethanol obtained was 1.17 g/L produced by acid pretreated samples. Harun and Danquah [31] in their research study obtained 1.0 g/L ethanol from acid pretreated (H_2_SO_4:_ 3% v/v) by loading 10 g of dried algae. On the other hand, Choi et al. [18] obtained 11.73 g/L bioethanol as highest yield without mentioning the quantity of biomass used (data not shown). In another research study conducted by Shokrkar et al. [36] 6.01 g/l ethanol was obtained by enzymatic hydrolysis (24 h) using 13.3 g/l sugars from dried microalgal biomass while the total ethanol yield was reduced from microalgal biomass obtained with acid hydrolysis (4.96 g/l ethanol from 13.05 g/l sugars: 0.5 M sulphuric acid).

**Table 3:** GC-MSD analysis of bioethanol produced by fermentation of alkaline, acid and enzyme pretreated samples of *Chorococcum sp*. TISTR 8583.

Pretreatment Chemical	Pretreatment Time	Ethanol Production (g/ 10 g dried algae)
NaOH (1.5%)	30min	1.40
H_2_SO_4_ (1%)	30min	1.17
Cellulase (0.015 g/ g)	72 hours	1.90

## 4 Conclusions

This study confirmed that *Chlorococcum* sp. *TISTR 8583* has the ability of biofuel production and accumulates high starch and lipid contents in nutrient stress conditions. Based on the strength of *Chlorococcum* sp. strains, the next generation fuel entrepreneurs may use it for production of good quality biodiesel (good quality FAME profile), high biomass and starch yield for its further utilization for value added products (bioethanol etc). The digestion of microalgal cell wall by different pretreatment methods facilitates the release of cytosolic starch for hydrolysis by amylase enzyme. The investigated microalgae strain was found to be a common platform for both bioethanol and biodiesel as compared to other biofuel producing bioresources. The acid and alkaline pretreatments are easily manageable and cost effective while the enzyme pretreatment is time consuming and relatively expensive. This study provides knowledge/understanding about the use of multiple strategies together for obtaining desired products such as enhancement experiment for sugars and lipids in investigated algal system followed by different pretreatment strategies and co-production of both fuel types (a single source for both biodiesel and bioethanol sequentially) using biorefinery concept and an insight into the impact of changing the physical (light intensity) and chemical conditions (nitrogen limitation) on the switched on/off of specific physiological pathways for obtaining enhanced yield of biomass and desired chemical compounds from algal systems for value added product development. Furthermore, this research study will provide knowledge to the readership about the production feasibility of sustainable and ecofriendly energy from these dynamic and versatile autotrophic aquatic creatures.

## Conflict of interest

None.

## Funding

This research did not receive any specific grant from funding agencies in the public, commercial, or not-for-profit sectors.
